# The Flavin-Containing Monooxygenase 3 Gene and Essential Hypertension: The Joint Effect of Polymorphism E158K and Cigarette Smoking on Disease Susceptibility

**DOI:** 10.1155/2014/712169

**Published:** 2014-08-27

**Authors:** Olga Bushueva, Maria Solodilova, Mikhail Churnosov, Vladimir Ivanov, Alexey Polonikov

**Affiliations:** ^1^Department of Biology, Medical Genetics and Ecology, Kursk State Medical University, Karl Marx Street, 3, Kursk 305041, Russia; ^2^Department of Medical Biological Disciplines, Belgorod State National Research University, Pobeda Street, 85, Belgorod 308015, Russia

## Abstract

Gene encoding flavin-containing monooxygenase 3 (*FMO3*), a microsomal antioxidant defense enzyme, has been suggested to contribute to essential hypertension (EH). The present study was designed to investigate whether common functional polymorphism E158K (rs2266782) of the *FMO3* gene is associated with EH susceptibility in a Russian population. A total of 2 995 unrelated subjects from Kursk (1 362 EH patients and 843 healthy controls) and Belgorod (357 EH patients and 422 population controls) regions of Central Russia were recruited for this study. DNA samples from all study participants were genotyped for the *FMO3* gene polymorphism through PCR followed by RFLP analysis. We found that the polymorphism E158K is associated with increased risk of essential hypertension in both discovery population from Kursk region (OR 1.36 95% CI 1.09–1.69, *P* = 0.01) and replication population from Belgorod region (OR 1.54 95% CI 1.07–1.89, *P* = 0.02) after adjustment for gender and age using logistic regression analysis. Further analysis showed that the increased hypertension risk in carriers of genotype 158KK gene occurred in cigarette smokers, whereas nonsmoker carriers of this genotype did not show the disease risk. This is the first study reporting the association of the *FMO3* gene polymorphism and the risk of essential hypertension.

## 1. Introduction

Oxidative stress resulting from the enhanced production of reactive oxygen species (ROS) and decreased activity of antioxidant defense enzymes has been implicated in pathogenesis of essential hypertension (EH) [1, 2]. The flavin-containing monooxygenase 3 (FMO3) is a microsomal antioxidant defense enzyme involving the NADPH-dependent oxygenation of a variety of nucleophilic xenobiotics possessing oxidant capacity [3]. Mutations in the* FMO3* gene have been found to contribute to the disease trimethylaminuria (TMAuria), an inborn error of metabolism resulting from diminished oxidation of the tertiary amine trimethylamine to trimethylamine N-oxide resulting in a severe body odour in affected individuals [4]. Interestingly, this genetic disorder is thought to be associated with risk of hypertension because patients with TMAuria have idiopathic hypertension [4, 5].

A single study investigated the relationship between common polymorphisms of the* FMO3* gene and hypertension in Irish population [6]. However, these researchers found that none of the* FMO3* gene polymorphisms was associated with hypertension risk. This negative result can be explained by a relatively low statistical power which is related with a small sample size in the study. The present study was designed to investigate whether common functional polymorphism E158K (rs2266782) of the* FMO3* gene is associated with susceptibility to essential hypertension in Russia. Pursuing this aim, two independent populations of ethnic Russians from Kursk and Belgorod regions of Central Russia were included in this study to be used as discovery and replication cohorts, respectively.

## 2. Materials and Methods

The study was approved by Ethical Review Committee of Kursk State Medical University. A total of 2 995 Russian unrelated subjects from Kursk (discovery cohort) and Belgorod (replication population) regions of Central Russia were included in the study. The discovery cohort comprising 2 216 subjects (1 362 EH patients and 843 healthy subjects with normal blood pressure) was recruited at Cardiology Clinics of Kursk Regional Clinical Hospital and Neurology Clinics of Kursk Emergency Medicine Hospital over two periods: between 2003 and 2006 [7] and between 2010 and 2013. The replication population included DNA samples from 779 individuals (357 EH patients and 422 population controls) which have been obtained from the biobank of Belgorod State National Research University, as part of a large population-based study of Belgorod region [8]. The baseline characteristics of the study patients are listed in Table 1. As can be seen from Table 1, hypertensive patients were matched to controls on sex and age (*P* > 0.05). Diagnosis of essential hypertension in both populations was verified by qualified cardiologists. Individuals were defined as hypertensive according to World Health Organization criteria or if they had a history of receiving any antihypertensive drug. Diagnosis of EH in untreated patients was defined by a seated systolic and/or diastolic blood pressure greater than 140 and/or 90 mmHg, respectively, on at least 2 separate measurements. All EH patients had no clinical signs, symptoms, and laboratory findings suggestive of secondary hypertension.

Genomic DNA was isolated from peripheral blood samples using a standard phenol/chloroform procedure. Genotyping of polymorphism E158K of the* FMO3* gene was done using PCR followed by RFLP analysis as described elsewhere [9]. The genotyping results were scored by two independent investigators blindly to the patient's case/control status and regenotyping of about 5% of randomly selected samples yielded 100% reproducibility.

The association between the polymorphism and hypertension risk was estimated by odds ratio (OR) with 95% confidence interval (CI) using multiple logistic regression analysis with adjustment for confounding variables such as age and gender. Each* FMO3* genotype was assessed according to dominant, recessive, and additive genetic models, and the chi-squire (Wald's statistic) odds ratio with 95% confidence interval was calculated. Odds ratios were calculated as a measure of the association of the* FMO3* genotype with hypertension risk, with the effects of the allele 158K assumed to be additive (with scores of 0, 1, and 2 assigned for EE, EK, and KK genotypes, resp.), dominant (with scores of 0 for EE genotype and 1 for EK and KK genotypes combined), or recessive (with scores of 0 for EE and EK genotypes combined and 1 for KK genotype). The statistical significance was established at* P ≤ *0.05. Bonferroni correction for *P* values (*P*_adj_) was applied in cases when multiple tests were performed. Statistical calculations were performed with Statistica for Windows 8.0 (StatSoft Inc., Tulsa, OK, USA).

## 3. Results and Discussion

As can be seen from Table 1, a percentage of positive family history of hypertension and cigarette smokers in the discovery cohort (these data were available only from Kursk population) was significantly greater in patients with EH versus healthy controls. Body mass index in hypertensives was higher than that in controls in both cohorts, but the difference in this parameter did not reach a statistical significance in Belgorod population (Table 1).

The* FMO3* genotype frequencies were in agreement with Hardy-Weinberg equilibrium (HWE) in control groups from both populations (*P* > 0.05). However, the genotype frequencies in hypertensive groups from both Kursk and Belgorod populations showed a deviation from HWE (*P* < 0.05) due to a decreased heterozygosity. Data on allele and genotype frequencies in Kursk and Belgorod populations are shown in Table 2. No significant difference in the allele 158K frequency was found between EH patients and controls in both cohorts. Allele and genotype frequencies were compatible with those reported previously in Kursk population [9]. Meanwhile, a statistically significant difference in the distribution of the* FMO3* genotypes was observed between the case and control groups of Kursk population (*P* = 0.01, df = 2). A statistically significant difference in the distribution of the* FMO3* genotypes between the case and control groups was also observed in Belgorod population (*P* = 0.02). The genotype 158KK was found to be associated with increased risk of hypertension in both Kursk and Belgorod populations after adjustment for gender and age using multiple logistic regression analysis.

Logistic regression analysis revealed that additive (Wald's chi-square = 1.81, *P* = 0.18) and dominant (Wald's chi-square = 0.09, *P* = 0.76) genetic models did not show a significant effect of the* FMO3* gene polymorphism on hypertension risk. Meanwhile, recessive model of the genetic association between the polymorphism and hypertension risk was established to be statistically significant (Wald's chi-square = 7.28, *P* = 0.007, *P*_adj_ = 0.02).

Data on the analysis for the* FMO3* genotype-cigarette smoking interaction and susceptibility to essential hypertension in Kursk population are shown in Table 3. The gene-smoking interaction analysis has revealed that genotype 158KK was associated with increased risk of hypertension only in smokers (OR = 1.38 95% CI 1.04–1.83, *P* = 0.023, *P*_adj_ = 0.046), whereas nonsmoker carriers of the genotype did not show the disease risk (OR = 1.25 95% CI 0.86–1.81, *P* = 0.24).

We can suggest two possible mechanisms by which the* FMO3* gene polymorphism contributes to the development of essential hypertension. Firstly, it is known that an amino acid substitution Glu-to-Lys at a position 158 (i.e., E158K polymorphism) is associated with decreased activity of the enzyme in oxidation of catecholamine releasing agents such as tyramine, phenylethylamine, adrenaline, and noradrenaline possessing vasopressor effects [6, 10]. This means that the low enzyme activity in carriers of the* FMO3* gene variant may be responsible for decreased catabolism of the vasoactive hormones, thereby contributing to hypertension risk through increased vasoconstriction and heart rate modulation. Secondly, a diminished activity of FMO3 in carriers of genotype 158KK may enhance ROS generation and induce oxidative stress, one of the proposed pathogenetic mechanisms of human hypertension [1, 2, 11–13]. Furthermore, we found that cigarette smoking strengthened the risk of essential hypertension in individuals with genotype 158KK. Notably, the effect of tobacco smoking on hypertension risk is mediated through the stimulation of the sympathetic nervous system [14], enhancing ROS generation and oxidative stress [15], that is, processes with which flavin-containing monooxygenase 3 is tightly related.

## 4. Conclusions

The present study demonstrated for the first time that the* FMO3* gene polymorphism is associated with susceptibility to essential hypertension, the result which was originally found in Kursk population and then validated in an independent population from Belgorod region. We also observed in the Kursk population that the increased risk of hypertension in carriers of heterozygous genotype 158KK of the* FMO3* gene occurs only in smokers, whereas nonsmokers possessing this genotype do not have the risk of the disease. This finding is an indication of the gene-environment interaction, a situation when adverse effects of cigarette smoking on hypertension can be enhanced by the phenotypic effects of the* FMO3* polymorphism. Although much remains to be learned regarding the biological basis of the relationship between the* FMO3* gene and hypertension risk, it is already clear that further studies focusing on the investigation of gene-environment interactions may improve our understanding of the disease pathogenesis and define novel therapeutic and preventive options as a means of personalized medicine in cardiologic practice.

## Figures and Tables

**Table 1:** Baseline characteristics of two study populations.

Baseline characteristics	EH patients	Controls	*P* values
Discovery cohort (Kursk region): 1 362 EH patients and 843 healthy subjects			
Age, mean ± standard deviation	56.4 ± 10.4	55.8 ± 8.8	0.18
Gender (M-male, F-female)	711 M (52.2%) 651 F (47.8%)	406 M (48.2%) 437 F (51.8%)	0.07
Body mass index (kg/m^2^), mean ± standard deviation	27.3 ± 6.5	26.2 ± 5.9	0.002*
Family history of hypertension	Yes 764 (65.9%) No 395 (34.1%)	Yes 414 (57.8%) No 302 (42.2%)	0.0004*
Smoking status	Yes 841 (67.4%) No 406 (32.6%)	Yes 483 (61.9%) No 297 (38.1%)	0.01*

Replication cohort (Belgorod region): 357 EH patients and 422 population controls			
Age, mean ± standard deviation	58.4 ± 9.5	57.3 ± 10.2	0.12
Gender (M-male, F-female)	211 M (59.1%) 146 F (40.9%)	267 M (63.3%) 155 F (36.7%)	0.23
Body mass index (kg/m^2^), mean ± standard deviation	26.2 ± 4.8	25.6 ± 3.9	0.07
Family history of hypertension	NA	NA	—
Smoking status	NA	NA	—

∗Means a significant difference between the groups.

**Table 2:** Allele and genotype frequencies for polymorphism E158K of the *FMO3* gene in patients with essential hypertension (EH) and controls in two Russian populations.

*FMO3 *allele and genotype frequencies	EH patients *N* (%)^I^	Controls *N* (%)^I^	Chi-square (*P*)^II^	OR (95% CI)
Discovery cohort (Kursk region), *N* = 2216 (1 362 EH patients and 843 healthy controls)				
Alleles				
158E 158K	1 474 (54.1) 1 250 (45.9)	960 (56.2) 748 (43.8)	1.86 (0.17) df = 1	1.09 (0.96–1.23)^III^
Genotypes				
158EE 158EK 158KK	423 (31.1) 628 (46.1) 311 (22.8)	260 (30.4) 440 (51.5) 154 (18.0)	9.02 (0.01)* df = 2	1.35 (1.08–1.69)^III^ 1.36 (1.09–1.69)^IV^

Replication population (Belgorod region), *N* = 779 (357 EH patients and 422 healthy controls)				
Alleles				
158E 158K	365 (51.1) 349 (48.9)	468 (55.5) 476 (44.5)	2.91 (0.09) df = 1	1.19 (0.97–1.45)^III^
Genotypes				
158EE 158EK 158KK	105 (29.4) 155 (43.4) 97 (27.2)	127 (30.1) 214 (50.7) 81 (19.2)	7.59 (0.02)* df = 2	1.57 (1.12–2.00)^III^ 1.54 (1.07–1.89)^IV^

^I^Absolute number and percentage of individuals with particular allele/genotype.

^II^Pearson's chi-square statistics and *P* values.

^III^Odds ratio (95% confidence interval) for association between 158KK genotype and EH risk.

^IV^Odds ratio (95% confidence interval) for association between 158KK genotype and EH risk adjusted for gender and age.

∗Means a significant association.

**Table 3:** The *FMO3* genotype cigarette smoking interaction and susceptibility to essential hypertension in Kursk population.

*FMO3* genotype	EH patients *N* (%)		Controls *N* (%)		OR (95% CI)	
	Smokers (*N* = 841)	Nonsmokers (*N* = 406)	Smokers (*N* = 483)	Nonsmokers (*N* = 297)	Smokers	Nonsmokers
158KK	198 (23.5)	93 (22.9)	88 (18.2)	57 (19.2)	**1.38** **(1.04–1.83)**∗	1.25 (0.83–1.81)

∗Means a statistically significant association (*P* = 0.023) after adjustment for multiple tests (*P*_adj_ = 0.046).

## References

[1] Briones AM, Touyz RM (2010). Oxidative stress and hypertension: current concepts. *Current Hypertension Reports*.

[2] Renna NF (2013). Oxidative stress, vascular remodeling, and vascular inflammation in hypertension. *International Journal of Hypertension*.

[3] Krueger SK, Williams DE (2005). Mammalian flavin-containing monooxygenases: structure/function, genetic polymorphisms and role in drug metabolism. *Pharmacology and Therapeutics*.

[4] Cashman JR, Akerman BR, Forrest SM, Treacy EP (2000). Population-specific polymorphisms of the human FMO3 gene: significance for detoxication. *Drug Metabolism and Disposition*.

[5] Akerman BR, Lemass H, Chow LML (1999). Trimethylaminuria is caused by mutations of the FMO_3_ gene in a North American cohort. *Molecular Genetics and Metabolism*.

[6] Dolan C, Shields DC, Stanton A (2005). Polymorphisms of the flavin containing monooxygenase 3 (*FMO3*) gene do not predispose to essential hypertension in Caucasians. *BMC Medical Genetics*.

[7] Polonikov AV, Ivanov VP, Solodilova MA (2008). A common polymorphism G-50T in cytochrome P450 2J2 gene is associated with increased risk of essential hypertension in a Russian population. *Disease Markers*.

[8] Sorokina IN, Churnosov MI, Balanovskaia EV (2009). The gene pool of the Belgorod region population: description of the “genetic landscape” of 22 district populations. *Genetika*.

[9] Vialykh EK, Solodilova MA, Bushueva OY, Bulgakova IV, Polonikov AV (2012). Catalase gene polymorphism is associated with increased risk of cerebral stroke in hypertensive patients. *Zhurnal Nevrologii i Psihiatrii imeni S.S. Korsakova*.

[10] Treacy EP, Akerman BR, Chow LML (1998). Mutations of the flavin-containing monooxygenase gene (*FMO3*) cause trimethylaminuria, a defect in detoxication. *Human Molecular Genetics*.

[11] Touyz RM (2000). Oxidative stress and vascular damage in hypertension. *Current Hypertension Reports*.

[12] de Champlain J, Wu R, Girouard H (2004). Oxidative stress in hypertension. *Clinical and Experimental Hypertension*.

[13] Vaziri ND, Rodríguez-Iturbe B (2006). Mechanisms of disease: oxidative stress and inflammation in the pathogenesis of hypertension. *Nature Reviews Nephrology*.

[14] Virdis A, Giannarelli C, Neves MF, Taddei S, Ghiadoni L (2010). Cigarette smoking and hypertension. *Current Pharmaceutical Design*.

[15] Talukder MAH, Johnson WM, Varadharaj S (2011). Chronic cigarette smoking causes hypertension, increased oxidative stress, impaired NO bioavailability, endothelial dysfunction, and cardiac remodeling in mice. *The American Journal of Physiology—Heart and Circulatory Physiology*.

